# Resolutions

**Published:** 1920-03

**Authors:** 


### RESOLUTIONS

These resolutions were prepared at the meeting of the American Institute of Dental Teachers, held at Detroit, Mich., in January:

### B. HOLLY SMITH, M.D., D.D.S.

For the second time on the eve of this meeting we are called upon to record the passing away of one of our esteemed members. Dr. B. Holly Smith has answered the final roll call. He passed away at his home in Baltimore, Saturday, January 24th, very suddenly.

It is impracticable on such short notice to do justice to the career of such a man, had we the power to put into words the thoughts that come surging into our memory.

We are first of all depressed with feelings of deep emotion that our friend has passed from our sight forever and we shall never meet his cheerful and gracious hand grasp and smiling countenance. To those who knew of his great devotion to his friends the shock is so great that it seems unreal and impossible.

To the profession of dentistry in all its departments there will be a vacant sphere of activity hard to supply. He was a man of varied capacities and because of his great interest in all dental affairs and because of his intense devotion to the cause he has for many years been identified with every professional activity of moment. His great zeal and indomitable will, together with his intellectual acquirements, made him unusually resourceful and capable. He served his profession in almost every conceivable capacity with credit to himself and great benefit to the profession. His work is done and his record is made and much of it is a matter of history that can be had from our published archives, but who can record the fraternal and spiritual contacts we have experienced as he has come and gone among us?

What these relations have done for us can never be known, as they are so fugitive as not to be recognized or tabulated. We are conscious of these influences, but we never stop to analyze them; nevertheless they exert a tremendous influence on the works that we feel are of greatest significance. We enjoy our friends and too often think of these contacts with them as of an accidental significance.

In the case of our friend, these incidents are numerous, and every member of this body will have some memory that has helped him and made his way easier and more cheerful and so helpful.

Our hearts are full of gratitude to-day that it was our good fortune to have been his friends. We value above price such friends and our loss when he passed away is greater than we can realize.

His loss to us is a real and serious one and we know it must be more serious to those who are near to him in the work which occupied his daily efforts.

The faculty of his school, his patients, the large circle of social acquaintances, and the hundreds of students who have received their training and inspiration from him, will miss his cheering words of counsel. They have suffered a great loss, but like the rest of us the works which he did for them are a great comfort and memory which will extend his life long into the future. He has passed from us, but is not dead; he lives in spirit and still helps in the great work to which he gave his time and effort in life.

We honor and revere him to-day for what he has been to us, though we have no adequate words; our hearts are sad and warm with affectionate esteem for our departed friend.

To his dear ones we extend our heartiest sympathy and pray for the comfort of the greatest of all Comforters.

### HENRY W. MORGAN, M.D., D.D.S.

The telegram announcing the death of Henry W. Morgan carried a message of deep regret and great sorrow to a host of admiring and sympathetic friends. His serious illness of many months had prepared those who were nearest to him for the announcement.

The passing of such a man from a life of such phenomenal activity and usefulness is always a shock and seems calamitous. In this case it was both. We are overcome by our personal loss and are anxious for the cause which he so ably served.

Dr. Morgan's life and career were no accident, but seem rather to have been foreordained. We can well believe he was born and bred for a life of extraordinary usefulness. His parents were well fitted to prepare him for the great work he has done and the inspiration he received from them doubtless kept him at his vocation with a steadfast devotion all these years. His father, a great student and leader, no doubt was a source of comfort and strength. His mental and moral endowments and his great interest in the cause of helping to make the world better and happier were in cordial sympathy with the work assumed by his son when he was forced to lay it down.

With such an inheritance we can readily see that Henry Morgan started his life's work with a momentum that is rare and a birthright which he treasured and lived up to with a conscientiousness that gave him initiative with an equilibrium that was the continued admiration of his friends.

We haven't time here to recount all that the career of Henry Morgan has given to the dental profession. He engaged most actively in every important matter of concern to the profession and was always ready to do his part. It may be enough to say that he always did his part faithfully and cheerfully, and that he always did it well. He accepted a call to duty as a trust, and never sought a place for reward or an honorarium.

He was distinctly a man of character. He did his own thinking and he was an earnest and thoughful student. He was ever open to enlightenment and to sensible and wholesome convictions. He was a big-hearted and tenderely affectionate brother; he loved mankind and men loved him; he was as gentle and kind as he was good, but he was courageous and determined for truth, which was fundamental to his very existence.

We have seen him contending with all his might for what he believed was true, but never have we known him to try to gain his purpose by intrigue. Justice and equity were not meaningless terms to him. It was a real pleasure for him to compliment any one by a fair concession.

It would be interesting, perhaps, if we could know how much money he paid out for traveling back and forth to dental meetings, and how much time he lost while engaged in such work. He undoubtedly realized the cost, but he never complained because it was a most important part of his life work—he just had to do it. The enormous drain on his physical strength by this effort makes a still larger debt which the dental profession and humanity in general can never repay.

He was a man of culture, and dentistry did not absorb him completely, as he had a large circle of humanitarian interests outside of our profession. He was a good citizen and kept in close touch with the political affairs of his own city as well as his state and the nation.

His interest in the benevolence of his church absorbed much of his time. He was an officer in Walden University and Maharry College for Colored Medical and Dental Students.

When the National School of Dental Technics was organized, Dr. Morgan was present and greatly interested. He made the motion when the constitution and by-laws were adopted at the second meeting that all members of faculties that were members in good standing in the National Association of Dental Faculties should be eligible to take part in the proceedings of this body. This right has never been questioned. This wise provision has held this body together all these years and made it the most democratic organization in our profession. We shall be wise to keep this institute free from all entangling alliances and an open forum for the discussion of our teaching problems. Dr. Morgan has always had a deep interest in the work of this body, and, if we are not mistaken, a faithful attendant at every meeting of this body since its organization. He is not with us to-day except in the spirit; he is, however, here to-day more forcibly than ever before. Every member of this body knew and loved him and was glad to work with him, and we all miss his glad hand to-day, but he is with us nevertheless and each one of us is glad to be here and communicate our innermost spiritual greeting as we have so often done in the body.

Our dear friend has finished his work here, but he will never be forgotten by those who knew and loved him. He will perhaps be forgotten by those who came after us, but his works will stand and the records will be kept, and the influences which he did so much to inaugurate and keep in motion will go on with increased power and by innumerable ways to more and more perfect attainments.

We are tempted to erect some enduring memorial to our friend that may outlast the memory, very much as the Disciples of Jesus wanted to do, but he would not allow them to do so. Our friend did deeds that will keep his memory dear to those of us who knew him, and the good influences of his busy and well-spent life will carry over through generations to come the eternal verities which made him the friend and power for good he was to those with whom he lived.

I am sure it is the universal desire of all the members of this institute that our sincere sympathy be extended to his family in their great bereavement. We trust his dear ones may have the supreme consolations of the Great Comforter and we cordially and sympathetically extend the assurance of our deep fraternal esteem.

